# Deciphering high risk molecular alterations in gastrointestinal malignancy utilizing an extreme outlier strategy

**DOI:** 10.18632/oncoscience.503

**Published:** 2020-05-05

**Authors:** Austin R. Dosch, Siddharth Mehra, Nipun B. Merchant, Jashodeep Datta

**Affiliations:** ^1^Division of Surgical Oncology, Dewitt Daughtry Department of Surgery, University of Miami Miller School of Medicine, Sylvester Comprehensive Cancer Center, Miami, FL, USA

**Keywords:** extreme outlier, gastrointestinal cancer, genomic, prognosis, high risk

The widespread adoption of next-generation sequencing (NGS) technologies has enabled cancer physicians and researchers alike to gain profound insight into the molecular underpinnings of malignant tumors, allowing appreciation of the heterogeneity in disease pathogenesis and its dependency on distinct genetic alterations [1]. This molecular understanding is particularly relevant in patients with gastrointestinal (GI) cancers, where outcomes are relatively dismal with standard multimodality cancer treatment (e.g., surgery, chemotherapy, radiotherapy, etc.) compared with many other solid cancers [2]. However, although the clinical heterogeneity of many GI cancers (e.g., metastatic colorectal cancer [CRC], gastric cancer, etc.) is well studied, the molecular basis of this variability remains poorly understood. To this end, several groups have attempted to navigate this genotype-phenotype chasm by utilizing pivotal genomic drivers [3] and gene expression classifiers [4, 5] to define molecular high-risk phenotypes. However, based on what we are continually learning about the molecular landscape of GI malignancy, these platforms provide a relatively myopic understanding of the picture.

In an effort to address this issue, our group has attempted to decipher high-risk molecular subtypes in GI malignancy using a novel *extreme outlier* strategy, highlighted in two recently published manuscripts [6, 7]. In this approach, extremes of a defined clinical outcome (e.g., extremely long survival in metastatic disease, unexpected early recurrence and death in very early-stage tumors, etc.) are selected, and the molecular characteristics underlying these extremes are investigated. In our opinion, this strategy alleviates the considerable problem of deciphering high-risk patient subgroups in *unselected* populations, a strategy that is often underpowered and its effects diluted by the frequent inclusion of patients yet to reach important outcome milestones. As such, an extreme outlier approach is decidedly advantageous in disease settings where the outcome of interest is observed infrequently, such as disease-related death in completely resected early gastric cancer or extraordinary >10-year survival in patients with metastatic colorectal cancer.

In the first manuscript, published in *Clinical Cancer Research* [6], we sought to identify the genomic underpinnings associated with extremes of survivorship following complete CRC metastasectomy, and validated these findings in two large independent cohorts (n=935, 443) of metastatic CRC patients. In the extreme outlier cohort, patients who underwent complete resection of colorectal liver metastases (CRLM) were stratified into groups based on extraordinarily long (≥10-year) or unexpected poor (≤2-year) overall survival. Upon analysis with a targeted exome capture NGS assay, although individual gene alterations were not prognostic of overall survival, concurrent mutations in both *KRAS* and *TP53* were significantly more likely to be present in ≤2-year survivors, whereas co-altered *KRAS-TP53* was absent in ≥10-year survivors (67% vs. 0%, P<0.001). When validated in two large cohorts of metastatic CRC, expansion of oncogenic *KRAS* mutations to encompass mutations in any Ras/Raf signaling pathway member (i.e., *KRAS*, *NRAS*, or *BRAF*) and their co-alteration with oncogenic *TP53* alterations was associated with significantly worse survival compared with alterations in either gene group alone. As such, three distinct prognostic clusters emerged in this analysis: (1) *TP53*-altered alone (median survival 132 months); (2) Ras/Raf-altered alone (65 months) or Ras/Raf- and *TP53* pan-wildtype (60 months); and (3) co-altered Ras/Raf-*TP53* (40 months; P<0.0001). Moreover, co-altered Ras/Raf-*TP53* was independently associated with mortality (HR 2.47, 95% CI 1.91-3.21, P<0.001). Taken together, these data suggest that molecular prognostication in metastatic colorectal cancer should extend beyond the isolated contributions of *KRAS*, *BRAF*, or *TP53* to a model inclusive of all these genetic elements. This molecular clustering was then leveraged to demonstrate its prognostic discrimination in patient subsets with distinct patterns of organ-specific metastasis—co-altered Ras/Raf-*TP53* was associated with worse survival in patients with liver and lung, but not peritoneal surface, metastasis. The more frequent presentation of co-altered Ras/Raf-*TP53* tumors with extrahepatic metastasis, particularly to sites with limited opportunities for therapeutic salvage (e.g., peritoneal surface, bone, brain, etc.) may suggest a putative molecular basis for the clinical heterogeneity observed in metastatic colorectal cancer. Conversely, the lack of prognostic discrimination offered by this genomic clustering in patients with peritoneal surface metastasis suggests the need for alternative biomarkers in this disease setting. Quite interestingly, convergence of the p53 and RTK-RAS pathways in the regulation of cellular fate has been known for years [8]; however, our recent study—utilizing the extreme outlier methodology framework—is the first to assert its biologic relevance in the clinical arena in CRC patients.

Our group has applied the extreme outlier paradigm in deciphering high-risk subgroups in gastric cancer as well, recently reported in the *British Journal of Surgery* [7]. In this study, we asked the simple question whether a distinct genomic profile could be associated with unexpected poor survival in patients with early gastric cancer (EGC) undergoing margin-negative gastrectomy? Utilizing an extreme outlier strategy, we examined a cohort of 263 patients to demonstrate that *TP53* hotspot mutations co-occurrent with loss of heterozygosity (TP53^MUT/LOH^) was significantly more frequent in resected EGC patients experiencing early death and was associated with worse disease-specific survival in a cohort of gastric cancer patients demonstrating extremes of survivorship following gastrectomy. This profile appeared specific to poor survival in early disease, as TP53^MUT/LOH^ was not prognostic in patients with locally advanced or metastatic gastric cancer. These data suggested that TP53^MUT/LOH^ may be a novel biomarker of poor survival in EGC and that this high-risk subgroup may deserve heightened surveillance and/or consideration of adjuvant or neoadjuvant therapies.

An unavoidable flaw of this extreme outlier approach is that it is somewhat reductionist, and may render a molecular picture that belies the true complexity of the heterogeneous tumor microenvironment. Genomic studies have proven that certain initiating mutations (i.e., *KRAS* mutations in pancreatic cancer) are virtually indispensable to the process of oncogenic transformation. These driver mutations often activate cellular processes that favor rapid proliferation coupled with loss of DNA-repair fidelity. Due to these properties, subsequent tumor cell progeny can accumulate additional mutations with extraordinary diversity, leading to the emergence of genetically distinct sub-clones within the same tumor. Additionally, selective pressures applied to the tumor microenvironment either by the host immune system (i.e., immunoediting) or by extrinsic treatments (i.e., chemotherapy, targeted therapies, immunotherapy) can lead to the genesis of more resistant cell populations with discrete genetic mutations that favor resistance [9]. Thus, the extreme outlier approach may oversimplify the genomic/transcriptomic heterogeneity depending on the subclone that is sampled, when in the disease course the tumor is sampled, or which prior systemic therapies patients have received. The recent adoption of single-cell DNA and RNA sequencing technologies may help to provide more insight into the heterogeneous molecular landscape of GI cancers and be utilized to dynamically evaluate potential prognostic mutations/gene signatures at a much higher cellular “resolution” than standard bulk sequencing approaches. However, concerns still remain with regards to these new technologies due to potential issues with tumor sampling as well as their relatively high cost.

**Figure 1:**
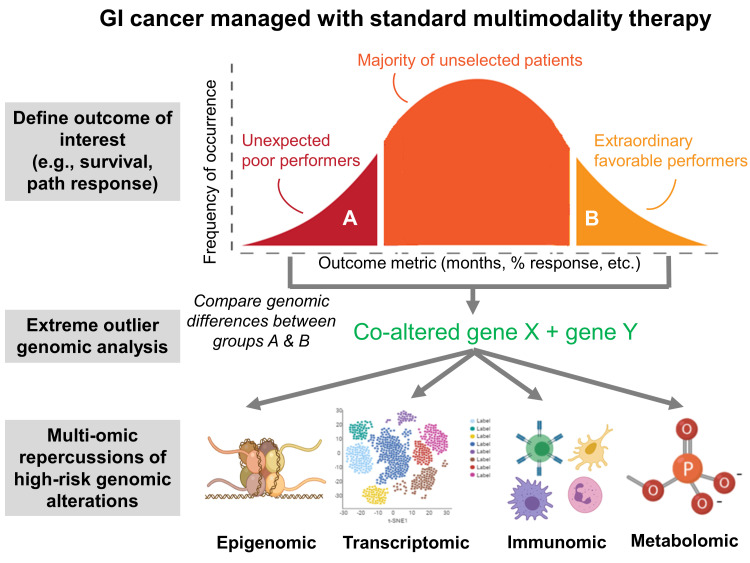
Schematic depicting the investigative journey from extreme outlier phenotypes to identification of underlying genotype to discovery of multi-omic repercussions of high-risk cooperative genomic alterations in gastrointestinal (GI) malignancies. The latter will represent targets for therapy in previously “undruggable” tumors with high-risk genomics.

While this extreme outlier strategy is indeed novel, and the high-risk molecular subgroups defined in the aforementioned studies prognostically meaningful, these molecular targets are not yet therapeutically actionable—both*KRAS* and *TP53* mutations are considered “undruggable.” The authors believe that unlocking the keys to targeting tumors with these cooperative molecular alterations (*KRAS-TP53, TP53*^MUT/LOH^, etc.) will require comprehensive understanding of their downstream (i.e., transcriptomic, immunomic, metabolic, etc.) or upstream (i.e., epigenomic) cellular consequences (Figure 1). Interestingly, McMurray and colleagues published an *in vitro* interrogation of Ras-p53 cooperativity in colon cancer cell lines which revealed a synergistic transcriptional program termed “cooperation response genes.” Of these candidates, several have since been identified as immunomodulatory chemokines [10]. In fact, our group is currently exploring the immune repercussions of these high-risk cooperative molecular alterations in the gastrointestinal tumor microenvironment. The ultimate goal is to establish a paradigm whereby targeting inhibitory immune consequences of cooperative high-risk genomic alterations may enhance susceptibility to immunotherapy and improve clinical outcomes in patients with these difficult-to-treat GI malignancies.
